# Characteristics of and Virulence Factors Associated with Biofilm Formation in Clinical *Enterococcus faecalis* Isolates in China

**DOI:** 10.3389/fmicb.2017.02338

**Published:** 2017-11-24

**Authors:** Jin-Xin Zheng, Yang Wu, Zhi-Wei Lin, Zhang-Ya Pu, Wei-Ming Yao, Zhong Chen, Duo-Yun Li, Qi-Wen Deng, Di Qu, Zhi-Jian Yu

**Affiliations:** ^1^Department of Infectious Diseases and Shenzhen Key Laboratory for Endogenous Infection, Shenzhen Nanshan People’s Hospital of Shenzhen University, Shenzhen, China; ^2^Key Laboratory of Medical Molecular Virology of Ministries of Education and Health, School of Basic Medical Science and Institutes of Biomedical Sciences, Shanghai Medical College of Fudan University, Shanghai, China

**Keywords:** *Enterococcus faecalis*, biofilm formation, virulence genes, multilocus sequence typing, linezolid resistance

## Abstract

*Enterococcus faecalis* biofilm traits and distribution characteristics in China have not been clarified. This study aimed to determine the prevalence and characteristics of *E. faecalis* biofilm formation in a sample of clinical isolates and to explore the virulence factors associated with biofilm formation in those isolates. A total of 265 *E. faecalis* isolates were collected from patients in Shenzhen, China. Virulence genes were detected within the genomes of the microbes by polymerase chain reaction. The isolates were subjected to multilocus sequence typing (MLST) based on housekeeping genes. Biofilms were detected by crystal violet staining. The expression levels of the clinical *E. faecalis* isolates’ genes were determined by quantitative real-time polymerase chain reaction. The prevalence of biofilm formation among *E. faecalis* clinical isolates was 47.2%. MLST yielded 44 different sequence types (STs). The main STs were ST16 and ST179; the ST16 isolates were more likely to form strong or medium biofilm than the ST179 isolates (*p* < 0.001). Strong or medium biofilm formation was more common in linezolid-resistant isolates than in linezolid-sensitive isolates (*p* = 0.001). Biofilm formation was more frequently detected in enterococcal surface protein (*esp+*), surface aggregating protein (*asa1*+), cytolysin A (*cylA*+), or aggregation substance (*agg*+) positive isolates than in isolates that were negative (-) for these virulence factors. Multivariate regression analysis indicated that *cylA* [odds ratio (OR) 4.083, *p* < 0.001] was a risk factor for weak biofilm formation, and that *esp* (OR 8.207, *p* < 0.001) was a risk factor for strong or medium biofilm formation. The expression of *cylA* was raised (4.02 to 6.00-fold) in weak biofilm isolates compared to the biofilm-negative isolates, and the expression of *esp* was greatly elevated (11.39 to 134.08-fold) in strong biofilm isolates compared to biofilm-negative isolates. In conclusion, the ST16 classification and linezolid resistance were positively associated with strong/medium biofilm formation in clinical *E. faecalis* isolates. *cylA* was associated with weak biofilm formation, and *esp* was only associated with strong or medium biofilm formation of the clinical *E. faecalis* isolates.

## Introduction

Enterococci are normal gut microbes in humans and other animals (Sghir et al., 2000; Damborg et al., 2009). However, in the last two decades, enterococcus pathogens have emerged as a major cause of nosocomial infections affecting various tissues, including the urinary tract, respiratory tract, peritoneum, and bloodstream (Treitman et al., 2005; Bonten and Willems, 2012). The most prevalent species cultured from humans, *Enterococcus faecalis* and *Enterococcus faecium*, account for more than 90% of clinical enterococcal isolates (Fisher and Phillips, 2009). It is noteworthy that *E. faecalis* and *E. faecium* cause treatment to be increasing difficult because of their intrinsic and acquired resistance to many antibiotics. *E. faecalis* and *E. faecium* have resistance to many commonly used antimicrobial agents, such as ampicillin and vancomycin (Van Harten et al., 2017). VRE especially have emerged as a major cause of outbreaks of nosocomial infection, which with their extensive resistance to a plethora of antibiotics have attracted more and more attention in recent years (Flokas et al., 2017). Linezolid, as the first antimicrobial agent of the oxazolidinones class, was widely used to treat VRE infections. Unfortunately, growing cases of linezolid-resistant enterococci have emerged in hospitals (Gawryszewska et al., 2017).

In addition to drug resistance, enterococci usually with a high capacity of biofilm formation, which has increased the difficulty of treatment. In Italy, 86.7% of *E. faecalis* clinical isolates formed different levels of biofilms, whereas only 15.6% of *E. faecium* isolates formed biofilms (Dupre et al., 2003). Among 109 enterococcal bloodstream isolates from Britain, 100% of *E. faecalis* strains had the ability to form biofilm, but only 42% of *E. faecium* isolates formed biofilms (Sandoe et al., 2003). *E. faecalis* isolates from the USA also had a high capacity of biofilm formation, Mohamed et al. found 93% of 163 *E. faecalis* isolates from various sources classified as biofilm producers (Mohamed et al., 2004). Another study from Japan indicated 100% of 352 *E. faecalis* isolates in urinary tract infections with different levels of biofilm formation (Seno et al., 2005). However, only 57.2% of *E. faecalis* isolates from Spain had the ability to form biofilms, and the 39 *E. faecium* isolates in the study couldn’t produce biofilms (Toledo-Arana et al., 2001). These studies found the *E. faecalis* isolates to have a higher capacity of biofilm formation than the *E. faecium* isolates, and the prevalence of *E. faecalis* biofilm differs regionally. However, up to now, the prevalence and characteristics of *E. faecalis* biofilm in China have remained obscure.

The phylogenetic relationships of pathogenic bacteria, including *Staphylococcus* spp. and *Enterococcus* spp., have been examined with MLST (Urwin and Maiden, 2003), a methodology that has yielded STs with distinctive virulence and drug resistance strain profiles (Manning et al., 2009). Biofilm formation has also been shown to vary across isolate STs (Kozitskaya et al., 2005). ST27 of *Staphylococcus epidermidis*, which is prone to biofilm formation, was found to occur preferentially in hospitals and to differ from clones in the community (Kozitskaya et al., 2005). The ST17 and ST19 lineages of group B *Streptococcus* strains isolated from invasive disease case samples were found to form weak biofilms relative to strains recovered from individuals with asymptomatic colonization (Parker et al., 2016). The biofilm formation properties of different STs of *E. faecalis* have not been characterized.

Previous researchers have reported that some virulence factors play important roles in the pathogenicity of *E. faecalis*, and several virulence factors may be related to *E. faecalis* biofilm formation. Esp is a large surface protein, which has been found to support cell adherence, colonization, and persistence in the urinary tract, evasion of the immune system, and to play an important role in *E. faecalis* biofilm formation (Toledo-Arana et al., 2001; Paganelli et al., 2012). GelE is an extracellular metalloprotease that hydrolyzes gelatin, collagen, and hemoglobin. GelE was responsible for regulating autolysis and the release of high-molecular-weight eDNA, an important component for the development of *E. faecalis* biofilms, and has also been reported to contribute to the bacterial adherence and biofilm formation of *E. faecalis* (Kayaoglu and Ørstavik, 2004; Park et al., 2007; Thomas et al., 2008). However, some research groups have found no significant correlation between the presence of Esp or GelE and *E. faecalis* biofilm formation (Kristich et al., 2004; Mohamed and Murray, 2005; Anderson et al., 2016).

Another virulence factor of *E. faecalis*, the Asa1, mediates aggregation between bacteria and enables the transfer of plasmids, was also found to promote biofilm formation in one study (Süßmuth et al., 2000; Chuang-Smith et al., 2010). The *cylA* gene of *E. faecalis* synthesizes a protein involved in the activation of cytolysin, and the lytic action of cytolysin on various cell types has been explored, including its contribution to the virulence of *E. faecalis* in infections (Van Tyne et al., 2013). Another study found *cylA* may be associated with *E. faecalis* biofilm formation in urinary tract infections (Seno et al., 2005). However, evidence of the involvement of *asa1* and *cylA* in *E. faecalis* biofilm formation is still deficient and needs to be confirmed by further studies. Hence, the virulence factors that enable *E. faecalis* biofilm formation remain ambiguous and controversial.

The aim of the present study was to explore the prevalence and characteristics of *E. faecalis* biofilm, and to identify virulence factors associated with *E. faecalis* biofilm formation. To our knowledge, this is the first study to combine data on biofilm formation, virulence genes, antibiotic resistance, and MLST of *E. faecalis* from China.

## Materials and Methods

### Bacterial Isolates

A total of 265 clinical *E. faecalis* isolates were obtained from patients at Shenzhen Nanshan Hospital, Shenzhen University in China between January 1 2010 and June 30 2016. Information on the antibiotics used in each case was collected from the electronic medical records database. Antibiotic use was as follows: 44 cases involved penicillins (ampicillin, piperacillin, amoxicillin), 15 cases involved glycopeptides (vancomycin, teicoplanin), 4 cases involved linezolid, and 37 cases involved other antibiotics (ciprofloxacin, levofloxacin, moxifloxacin, minocycline). The strains isolated from the patients were identified with a VITEK 2 microbial testing system (bioMérieux, Marcy l’Etoile, France). *E. faecalis* ATCC29212 and OG1RF (ATCC47077) strains purchased from the American Type Culture Collection were used as reference strains. All procedures involving human participants were performed in accordance with the ethical standards of Shenzhen University and with the 1964 Helsinki declaration and its later amendments, or comparable ethical standards. For this type of study, formal consent is not required.

### Antibiotic-Susceptibility Testing

The susceptibilities of the isolates to clinically relevant antibiotics (i.e., penicillin G, ampicillin, imipenem, ciprofloxacin, levofloxacin, teicoplanin, vancomycin, tigecycline, cotrimoxazole, linezolid, high-level gentamicin, erythromycin, and tetracycline) were determined with the VITEK 2 system (bioMérieux, Marcy l’Etoile, France). We determined the MICs of ampicillin, vancomycin, and linezolid using the broth macrodilution method according to CLSI guidelines, with CLSI-recommended MIC breakpoints.

### Isolation of DNA

DNA was extracted from isolates and purified with a DNeasy Blood and Tissue Kit (Qiagen China Co., Ltd., Zhangjiang Hi-Tech Park Pudong, Shanghai, China) according to the manufacturer’s protocol for Gram-positive bacteria. The microbial DNA was eluted with 100 μl of AE buffer (Qiagen) and stored at -20°C.

### Detection of Virulence Genes by PCR

The extracted DNA served as a template for the amplification of virulence genes. All primer sequences and corresponding references are listed in **Table 1** (Vankerckhoven et al., 2004; Dupre et al., 2003). PCR amplification was performed in a total volume of 50 μl, containing 2× PCR Master Mix (Tiangen Biotech Beijing Co., Ltd., Beijing, China), 0.5 mM of each primer, and 1 μl template DNA. The cycling conditions were as follows: 95°C for 3 min; followed by 30 cycles at 95°C for 30 s, 52°C for 30 s, 72°C for 60 s; and a final 10 min extension step at 72°C. Each PCR set included a no-template control and a positive control. The amplification products were analyzed by electrophoresis in 1.0% agarose gels.

**Table 1:** PCR primers used for detection of *Enterococcus faecalis* virulence factors.

Target	Primer	Primer sequence (5′–3′)	Amplicon size (bp)	Reference
*esp*	*esp*-F	AATTGATTCTTTAGCATCTGG	510	Vankerckhoven et al., 2004
	*esp*-R	AGATTCATCTTTGATTCTTGG		
*gelE*	*gelE*-F	TATGACAATGCTTTTTGGGAT	213	Vankerckhoven et al., 2004
	*gelE*-R	AGATGCACCCGAAATAATATA		
*asa1*	*asa1*-F	GCACGCTATTACGAACTATGA	375	Vankerckhoven et al., 2004
	*asa1*-R	TAAGAAAGAACATCACCACGA		
*cylA*	*cylA*-F	ACTCGGGGATTGATAGGC	688	Vankerckhoven et al., 2004
	*cylA*-R	GCTGCTAAAGCTGCGCTT		
*hyl*	*hyl*-F	GACTGACGTCCAAGTTTCCAA	276	Vankerckhoven et al., 2004
	*hyl*-R	ACAGAAGAGCTGCAGGAAATG		
*agg*	*agg*-F	CCAGTAATCAGTCCAGAAACAACC	406	Dupre et al., 2003
	*agg*-R	TAGCTTTTTTCATTCTTGTGTTTGTT		

### Multilocus Sequence Typing

Multilocus sequence typing was conducted by the reference method (Ruiz-Garbajosa et al., 2006). Briefly, seven housekeeping genes (*gdh, gyd, pstS, gki, aroE, xpt*, and *yqiL*) were PCR amplified and sequenced. All primer sequences and corresponding references are listed in **Table 2**. The PCR system and cycling conditions were the same as indicated above for the virulence genes. For each locus, a distinct allele number was assigned to each unique sequence, in accordance with the *E. faecalis* MLST database^1^. The seven STs were assigned in the order *gdh, gyd, pstS, gki, aroE, xpt*, and *yqiL*, corresponding to the allele numbers at the seven loci. ST names were kept consistent as much as possible for the same strains.

**Table 2:** PCR primers used for *E. faecalis* MLST gene diversity determination.

Target	Primer	Primer sequence (5′–3′)	Amplicon size (bp)	Reference
*gdh*	*gdh*-F	GGCGCACTAAAAGATATGGT	530	Ruiz-Garbajosa et al., 2006
	*gdh*-R	CCAAGATTGGGCAACTTCGTCCCA		
*gyd*	*gyd*-F	CAAACTGCTTAG CTCCAATGGC	395	Ruiz-Garbajosa et al., 2006
	*gyd*-R	CATTTCGTTGTCATACCAAGC		
*pstS*	*pstS*-F	CGGAACAGGACTTTCGC	583	Ruiz-Garbajosa et al., 2006
	*pstS*-R	ATTTACATCACGTTCTACTTGC		
*gki*	*gki*-F	GATTTTGTGGGAATTGGTATGG	438	Ruiz-Garbajosa et al., 2006
	*gki*-R	ACCATTAAAGCAAAATGATCGC		
*aroE*	*aroE*-F	TGGAAAACTTTACGGAGACAGC	459	Ruiz-Garbajosa et al., 2006
	*aroE*-R	GTCCTG TCCATTGTTCAAAAGC		
*xpt*	*xpt*-F	AAAATGATGGCCGTGTATTAGG,	456	Ruiz-Garbajosa et al., 2006
	*xpt*-R	AACGTCACCGTTCCTTCACTTA		
*yqiL*	*yqiL*-F	CAGCTTAAGTCAAG TAAGTGCCG	436	Ruiz-Garbajosa et al., 2006
	*yqiL*-R	GAATATCCCTTCTGCTTGTGCT		

### Biofilm Assay

*Enterococcus faecalis* biofilm formation was detected according to the reference method, with some modifications (Toledo-Arana et al., 2001; Mohamed et al., 2004). Briefly, bacteria that had been grown overnight were diluted 1:100 in 200 μl of tryptic soy broth with 0.25% glucose and inoculated onto polystyrene microtiter plates (Costar3599; Corning). After 24 h of static incubation at 37°C, the supernatant was discarded, and plates were washed thrice with deionized water to remove unattached cells, then fixed with methanol for 30 min, stained with 1% crystal violet for 30 min, and rinsed with distilled water. The optical density at 570 nm (OD_570_) was determined. Each assay was performed in triplicate on at least three occasions.

### RNA Extraction and qRT-PCR

The expression levels of the *esp* and *cylA* genes of *E. faecalis* clinical isolates were determined by qRT-PCR. The primers used for qRT-PCR are listed in **Table 3**. The RNA extraction was carried out according to the reference method (Xu et al., 2017), with some modifications: the *E. faecalis* strains were cultured at 37°C until the OD_600_ reached 0.6. Then, the cell pellets were washed with ice-cold normal saline and then homogenized using 0.1 mm Zirconia-silica beads in a Mini-BeadBeater (Biospec, Bartlesville, OK, United States) at a speed of 4,000 rpm for 1 min, followed by cooling on ice for 1 min. This homogenization and cooling cycle was repeated five times; then, the samples were centrifuged at 15,000 rpm and the bacterial RNA in the supernatant was purified using an RNeasy Mini Kit (Qiagen) and quantified using an ND-1000 spectrophotometer (NanoDrop Technologies, Wilmington, DE, United States). RNA samples that had a 260/280 ratio between 2.0 and 2.2 were reverse transcribed with the PrimeScript RT Reagent Kit (TaKaRa). Finally, qRT-PCR was performed with the SYBR Premix Ex Taq II Kit (TaKaRa) on the Mastercycler ep realplex system (Eppendorf), with an initial incubation at 95°C for 2 min, followed by 40 cycles of 15 s at 95°C, and 60 s at 60°C. Each sample was analyzed in triplicate.

**Table 3:** PCR primers used for determining the expression levels of *esp* and *cylA* of *E. faecalis* isolates by quantitative real-time polymerase chain reaction (qRT-PCR).

Target	Primer	Primer sequence (5′–3′)	Amplicon size (bp)	Reference
*recA*	*recA*-F	CGACTAATGTCTCAAGCACTAC	106	This study
	*recA*-R	CGAACATCACGCCAACTT		
*esp*	*Resp*-F	GCATCAGTATTAGTTGGT	196	This study
	*Resp*-R	TTCCTTGTAACACATCAC		
*cylA*	*RcylA*-F	GGAGGATATGGTGACAAT	163	This study
	*RcylA*-R	TTACTTCTGGAGTTGCTAA		

For all samples, the internal control gene *recA* (Ruiz-Cruz et al., 2015) was used to normalize the expression of the *esp* and *cylA* genes. The threshold cycle (Ct) numbers were confirmed by the detection system software and the data were analyzed based on the 2^-ΔΔCt^ method.

### Statistical Analysis

The prevalence of *E. faecalis* biofilm is reported as a number (percentage) and was compared using the chi-square test or Fisher’s exact test. The virulence factors associated with biofilm formation were analyzed with a multivariate logistic regression model constructed by backward selection based on the Wald statistic. ORs are reported with 95% CIs. *P*-values < 0.05 were regarded as statistically significant. All data were analyzed using the statistical software SPSS (version 14.0; SPSS, Chicago, IL, United States).

## Results

### Biofilm Formation

OD_570_ microplate readings after crystal violet staining ranged from 0.05 to 3.5. The numbers and percentages of the isolates, altogether and segregated by clinical source, and the biofilm phenotype categorized based on the approach of others (Toledo-Arana et al., 2001; Mohamed et al., 2004), with a strong (OD_570_ > 2), medium (OD_570_, 1–2), or weak (OD_570_ > 0.5 and <1) biofilm formation phenotype, are reported in **Table 4**. The median OD_570_ values for control strains OG1RF [weak biofilm (Montealegre et al., 2015)] and ATCC29212 were 0.56 and 0.15, respectively.

**Table 4:** Occurrence of *E. faecalis* biofilm formation by clinical source.

Clinical source (no. isolates tested)	No. (%) of isolates with biofilm phenotype				
	Strong	Medium	*Strong or medium*	Weak	All positive
Blood (25)	5 (20.0)	1 (4.0)	*6 (24.0)*	4 (16.0)	10 (40.0)
Urine (113)	15 (13.3)	15 (13.3)	*30 (26.6)*	27 (23.9)	57 (50.4)
Pus or secretions (64)	6 (9.4)	1 (1.6)	*7 (11.0)*	22 (34.4)	29 (45.3)
Bile (19)	1 (5.3)	4 (21.1)	*5 (26.3)*	2 (10.5)	7 (36.8)
Other^a^ (44)	8 (18.2)	3 (6.8)	*11 (25.0)*	11 (25.0)	22 (50.0)
Total (265)	35 (13.2)	24 (9.1)	*59 (22.3)*	66 (24.9)	125 (47.2)

***^a^**Sputum, tissue, catheters, pleural effusion, ascites fluid, amniotic fluid, puncture fluid, cerebrospinal fluid, throat swabs.*

As reported in **Table 4**, biofilms were observed for nearly half of all of the *E. faecalis* isolates examined, and among these biofilm-forming strains, the majority were classified as having a weak biofilm phenotype. With respect to clinical source, isolates from urine were the most likely to exhibit biofilm formation, followed by isolates from bile; the likelihood of biofilm formation was statistically similar across the remaining sources.

### Association between ST and Biofilm Formation

Multilocus sequence typing of 265 *E. faecalis* clinical isolates enabled us to determine the STs for 224 isolates. We found 44 different STs, 41 of which were represented by four or fewer isolates. The biofilm characteristics and numbers of isolates assigned to each ST are reported in Supplementary Table S1.

Because ST16 and ST179 were quite dominant, accounting for 65.6% (147/224) of the isolates in this study, the biofilm formation characteristics of the ST16 and ST179 isolates were subjected to further analysis. As shown in **Table 5**, biofilm formation, and strong or medium biofilm formation in particular, were detected significantly more frequently among ST16 isolates than among ST179 isolates. Further analysis of the clinical sources of ST16 and ST179 *E. faecalis* isolates indicated that the characteristics of each were consistent across clinical sources (Supplementary Table S2).

**Table 5:** Biofilm-forming capacity comparison between ST16 and ST179 isolates.

Biofilm phenotype	No. (%) with phenotype		*P*
	*ST16, N* = 79	*ST179, N* = 68
Strong	19 (24.1)	1 (1.5)	<0.001
Medium	9 (11.4)	3 (4.4)	NS
*Strong or medium*	*28 (35.4)*	*4 (5.9)*	<0.001
Weak	20 (25.3)	24 (35.3)	NS
All positive	48 (60.8)	28 (41.2)	0.018

*NS, not significant.*

### Association between Biofilm Formation and Linezolid Susceptibility

Using the broth macrodilution method to determine the MICs of ampicillin, vancomycin, and linezolid, and a resistance criterion classification of MIC ≥8μg/ml, we found 10 strains with linezolid resistance (the 10 patients received no linezolid treatment), only four strains with ampicillin resistance (only 1 of the 4 patients received piperacillin-tazobactam treatment), and none with vancomycin resistance. Follow-up analysis of the relationship between *E. faecalis* biofilm formation and linezolid susceptibility showed that the linezolid-resistant strains were more likely to exhibit biofilm formation than the linezolid-sensitive strains (MIC ≤ 2 μg/ml), and that the biofilm formation characteristics of the isolates with a linezolid MIC in the intermediate range (2–8 μg/ml; *n* = 41) did not differ significantly from that of the lower or higher MIC isolate groups (**Table 6**). More specifically, strong or medium biofilm formation was more prevalent among linezolid-resistant isolates than among linezolid-sensitive isolates (**Table 6**). Two ST16 isolates and no ST179 isolates were included among the linezolid-resistant strains; biofilm prevalence did not differ significantly between linezolid-sensitive and -resistant isolates (Supplementary Table S3).

**Table 6:** Correlation between biofilm-forming capacity and linezolid sensitivity.

Biofilm phenotype	No. (%) with linezolid MIC in μg/ml		
	MIC ≤ 2, *N* = 214	MIC 2–8, *N* = 41	MIC ≥ 8, *N* = 10
Strong	24 (11.2)	9 (22.0)	2 (20.0)
Medium	19 (8.9)	0 (0.0)	5 (50.0)^a^
*Strong or medium*	*43 (20.1)*	*9 (22.0)*	*7 (70.0)^b^*
Weak	52 (24.3)	12 (29.3)	2 (20.0)
All positive	95 (44.4)	21 (51.2)	9 (90.0)^c^

***^a^**Medium: MIC ≥ 8 vs. MIC ≤ 2, *p* = 0.002. **^b^***Strong or medium*: MIC ≥ 8 *vs*. MIC ≤ 2, *p* = 0.001. **^c^**Positive: MIC ≥ 8 *vs*. MIC ≤ 2, *p* = 0.007.*

### Correlation between Biofilm Formation and Virulence Factors

As reported in **Table 7**, the analysis of PCR-amplified virulence factors showed that the *esp*-positive (+) *E. faecalis* isolates had a significantly greater prevalence of biofilm formation than the *esp*-negative (-) isolates. Additionally, biofilm formation was detected more frequently in *asa1*+, *cylA*+ (cytolysin A-encoding gene positive), and *agg*+ (aggregation substance-encoding gene positive) isolates than in isolates not expressing each of these virulence factors (**Table 7**). Conversely, *gelE*- isolates were significantly more likely to have biofilm formation than *gelE*+ isolates, especially biofilms categorized as strong or medium (**Table 7**, see Footnote).

**Table 7:** Relationship between biofilm-forming capacity and virulence factors.

Virulence factors (no. isolates tested)	No. (%) of isolates with biofilm phenotype					*P*^a^
	Strong	Medium	*Strong or medium*	Weak	All positive	
*esp*+ (163)	30 (18.4)	20 (12.3)	*50 (30.7)*	47 (28.8)	97 (59.5)	*<0.001*
*esp*- (102)	5 (4.9)	4 (3.9)	*9 (8.8)*	19 (18.6)	28 (27.5)	
*gelE*+ (171)	12 (7.0)	13 (7.6)	*25 (14.6)*	46 (26.9)	71 (41.5)	*0.013*
*gelE*- (94)	23 (24.5)^b^	11 (11.7)	*34 (36.2)^c^*	20 (21.3)	54 (57.4)	
*asa1*+ (210)	32 (19.4)	20 (9.7)	*52 (29.1)*	57 (26.0)	109 (55.1)	*0.003*
*asa1*- (55)	3 (5.5)	4 (7.3)	*7 (12.7)*	9 (16.4)	16 (29.1)	
*cylA*+ (178)	26 (14.6)	19 (10.7)	*45 (25.3)*	57 (32.0)	102 (57.3)	*<0.001*
*cylA*- (87)	9 (10.3)	5 (5.7)	*14 (16.1)*	9 (10.3)	23 (26.4)	
*hyl*+ (83)	7 (8.4)	6 (7.2)	*13 (15.7)*	20 (24.1)	33 (39.8)	0.103
*hyl*- (182)	28 (15.4)	18 (9.9)	*46 (25.3)*	46 (25.3)	92 (50.5)	
*agg*+ (88)	11 (12.5)	12 (13.6)	*23 (26.1)*	27 (30.7)	50 (56.8)	*0.027*
*agg*- (177)	24 (13.6)	12 (6.8)	*36 (20.3)*	39 (22.0)	75 (42.4)	

***^a^**Biofilm formation: + vs. –. **^b^**Strong biofilm: *gelE*– *vs*. *gelE*+, *p* < 0.001. **^c^**Strong or medium biofilm: *gelE*– *vs*. *gelE*+, *p* < 0.001.*

### Virulence Factor Association with Biofilm Formation by Multiple Regression Analysis

In order to determine the independent contribution of each virulence factor to the biofilm formation of *E. faecalis*, multiple logistic regression analysis was performed. Thus, *esp, gelE, asal, cylA*, and *agg* were used as independent variables, and weak biofilm formation, strong or medium biofilm formation were used as a dependent variable in the multivariate model. The logistic regression model was constructed by a backward selection approach based on the Wald statistic. *P*-values < 0.05 were regarded as statistically significant. As shown in **Table 8**, only one factor, *cylA*, was found to be an independent risk factor for weak biofilm formation. Meanwhile, *esp* positivity was independently associated with strong or medium biofilm formation, and *gelE* emerged as an independent anti-risk factor for strong or medium biofilm formation.

**Table 8:** Multivariate regression analysis of virulence factors associated with *E. faecalis* biofilm formation.

Isolate biofilm phenotype	Factor	OR (95% CI)	*P*
Weak biofilm formation			
	*cylA*	4.083 (1.912–8.716)	*<0.001*
Strong or medium biofilm formation			
	*gelE*	0.300 (0.159–0.564)	*<0.001*
	*cylA*	0.393 (0.146–1.056)	0.064
	*esp*	8.207 (2.795–24.099)	*<0.001*

### Expression Levels of the *esp* and *cylA* Genes in *E. faecalis* Clinical Isolates

**FIGURE 1:**
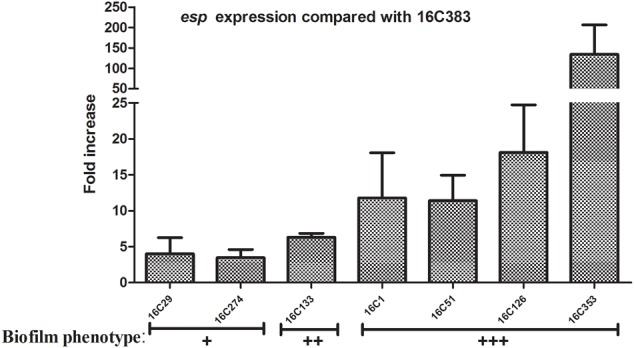
Relative fold expression of *esp* in seven clinical *Enterococcus faecalis* isolates. The expression levels of *esp* were determined by quantitative real-time polymerase chain reaction (qRT-PCR), with the clinical *E. faecalis* isolate 16C383 as the reference strain (expression = 1, biofilm negative). Biofilm phenotype: +, weak; ++, medium; +++, strong. The *esp* was overexpressed in the four strong biofilm strains (11.39 to 134.08-fold).

Last, in order to clarify the role of *E. faecalis* virulence factors during biofilm formation, the expression levels of the *esp* and *cylA* genes of *E. faecalis* clinical isolates were determined by qRT-PCR. For the detection of *esp* expression, eight clinical *E. faecalis* isolates that were *esp* positive, and with the same phenotypes of other virulence factors (*asa1*+; *gelE-*; *cylA-*; *hyl-*; *agg*+) were chosen. Among those 8 *esp*-positive isolates, the 16C383 strain with negative biofilm formation was set as the reference strain (*esp* expression = 1). As shown in **Figure 1**, the *esp* expression was markedly elevated (11.39 to 134.08-fold, respectively) in those strong biofilm isolates (16C1, 16C51, 16C126, and 16C353), compared with the weak biofilm producers (16C29, 4.01-fold and 16C274, 3.47-fold). These results suggest that *esp* was only associated with strong biofilm, not with the weak biofilm formation of *E. faecalis* isolates, consistent with the previous findings.

**FIGURE 2:**
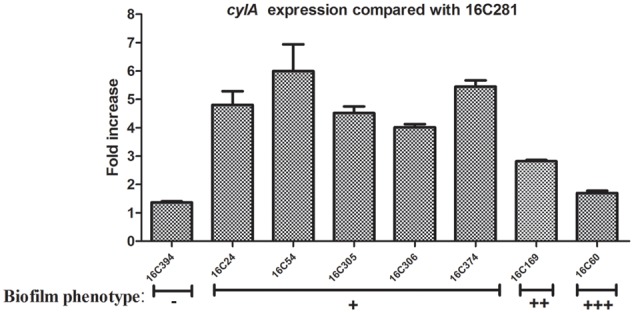
Relative fold expression of *cylA* in eight clinical *E. faecalis* isolates. The expression levels of *cylA* were determined by qRT-PCR, with the clinical *E. faecalis* isolate 16C281 as the reference strain (expression = 1, biofilm negative). Biofilm phenotype: –, negative; +, weak; ++, medium; +++, strong. The *cylA* was overexpressed in the five weak biofilm strains (4.02 to 6.00-fold).

For the test of *cylA* expression, nine clinical *E. faecalis* isolates that were *cylA* positive, and with the same phenotypes of other virulence factors (*asa1*+; *gelE-*; *esp-*; *hyl-*; *agg*+) were chosen. Among those 9 *cylA*-positive isolates, the 16C281 strain with negative biofilm formation was set as the reference strain (*cylA* expression = 1). As shown in **Figure 2**, the *cylA* gene expression was higher (4.02 to 6.00-fold) in those weak biofilm isolates (16C24, 16C54, 16C305, 16C306, and 16C374), compared with in the negative biofilm isolate (16C394, 1.37-fold), or the medium (16C169, 2.82-fold) or strong (16C60, 1.70-fold) biofilm isolates. These data may confirm that the *cylA* gene was only associated with weak biofilm, not with the strong or medium biofilm formation of *E. faecalis* isolates.

## Discussion

Although *E. faecalis* is generally less drug resistant than *E. faecium*, we found that biofilm formation was more prevalent for *E. faecalis* than has been reported previously for *E. faecium* (Sandoe et al., 2003). The prevalence of *E. faecalis* biofilm observed in this study, 47.2%, was lower than the 60–90% prevalence rates reported previously in Europe (Toledo-Arana et al., 2001; Dupre et al., 2003; Sandoe et al., 2003). In particular, the prevalence of *E. faecalis* biofilm in this study for isolates from urine, 50.4%, and from blood, 40.0%, were essentially half of the 100% prevalence rates reported previously for isolates from urine (Seno et al., 2005) and blood (Sandoe et al., 2003). Among the 57 *E. faecalis* isolates from urine with biofilm formation ability, 47.4% (27/57) of isolates were weak biofilm producers. Another study from Japan found in 352 *E. faecalis* strains from urinary tract infections that all of them had the capacity to form biofilm, 37.5% (132/352) of isolates exhibited weak biofilm formation (Seno et al., 2005). Our study found the *cylA* was associated with *E. faecalis* isolates with weak biofilm formation, and the prevalence of *cylA* in the biofilm-positive *E. faecalis* isolates from urine was 78.9% (45/57), which was higher than in the Seno et al. research (46.6%, 164/352). Thus, the prevalence of the weak biofilm phenotype in those *E. faecalis* isolates from urine may be due to the expression of *cylA*, based on the present study.

The lower rates of *E. faecalis* biofilm observed here could be due to several possible reasons. First, the *E. faecalis* strains isolated from different studies may have different ST frequencies. Second, because crystal violet staining analysis is a semi-quantitative method, it carries a risk of operation error. Last, there is not yet a consistent criterion for the determination of a positive biofilm, particularly with respect to a crystal violet optical density cut-off value.

The present demonstration of 44 STs provides, to the best of our knowledge, the first MLST of *E. faecalis* in China. The dominance of ST16 and ST179 among our samples is somewhat consistent with a prior study in Malaysia, in which ST6, ST16, ST28, ST179, and ST399 were represented (Weng et al., 2013). Our finding of greater biofilm prevalence, especially strong/medium biofilm prevalence, among ST16 isolates than among ST179 isolates is noteworthy in light of previous evidence showing that ST16 isolates may be representative of hospital-adapted strains (Ruiz-Garbajosa et al., 2006), and the observation that, relative to planktonic bacteria, biofilms enable bacteria to better survive in adverse environmental conditions, including in the presence of antibiotics and disinfectants (Lebeaux et al., 2014). *E. faecalis* ST16 may, like *S. epidermidis* ST27 (Kozitskaya et al., 2005), occur preferentially in nosocomial environments, with an atypically high prevalence of biofilm formation relative to other STs of the microbe species.

The relationship between *E. faecalis* biofilm formation and antibiotic resistance had not been well explored before this study. Interest in VREs has been growing in recent years (O’Driscoll and Crank, 2015). Linezolid has been used to treat VRE infection, but wider use of linezolid may be increasing the prevalence of linezolid resistance in *E. faecalis* (Kainer et al., 2007). Indeed, here we found ten *E. faecalis* isolates that were resistant to linezolid, which was with no linezolid treatment, but found no VRE isolates. Moreover, we found that the prevalence of biofilm formation, especially the strong or medium biofilm formation, was significantly higher in linezolid-resistant *E. faecalis* isolates than in linezolid-sensitive isolates. Although *E. faecalis* ST16 was found to have a high prevalence of biofilm formation, ST16 isolates were similarly represented across linezolid-sensitive and -resistant strains, indicating that biofilm formation by linezolid-resistant isolates cannot be attributed to the ST16 profile *per se*. Further research is needed to examine whether biofilm formation plays a role in *E. faecalis* resistance to linezolid.

The present finding that *esp*+ isolates are more likely to exhibit strong or medium biofilm formation than *esp-* isolates is consistent with several previous studies that have suggested a link between the virulence factor Esp and *E. faecalis* biofilm formation (Toledo-Arana et al., 2001; Tendolkar et al., 2004; Paganelli et al., 2012). Notwithstanding, other studies have failed to find evidence of such a link (Kristich et al., 2004; Mohamed and Murray, 2005; Di Rosa et al., 2006; Elhadidy and Elsayyad, 2013; Anderson et al., 2016). We did not observe an association between *esp* positivity and weak biofilm formation, suggesting the possibility that *esp* might *enhance* extant *E. faecalis* biofilm formation, without having a fundamental influence on the basic capacity for biofilm formation *per se*.

Our finding of *gelE* positivity being inversely associated with strong or medium biofilm formation in *E. faecalis* is surprising. Previous studies have suggested that the *gelE*-encoded protein, which can hydrolyze gelatin, collagen, and hemoglobin, may favor the development of *E. faecalis* biofilms (Kayaoglu and Ørstavik, 2004; Park et al., 2007; Thomas et al., 2008), while others found no significant correlation between the presence of *gelE* and biofilm formation in *E. faecalis* (Mohamed and Murray, 2005; Anderson et al., 2016). It might be that the STs of our *E. faecalis* strains differed from those of previous studies, or there may be as yet unidentified factors that determine *E. faecalis* biofilm formation.

Our negative finding with respect to *asa1* affecting biofilm formation is inconsistent with a prior study showing as association between *asa1* and *E. faecalis* biofilm formation (Chuang-Smith et al., 2010). Our finding that *cylA* was only a significant factor in weak biofilm formation suggests that *cylA* may be irrelevant to strong or medium biofilm formation in *E. faecalis*. The *cylA* gene has been reported previously to be associated with the *E. faecalis* biofilm formation of urinary tract isolates (Seno et al., 2005). However, thus far, there is little information regarding the potential role of *cylA* – which can lyse prokaryotic cells, as well as erythrocytes and other eukaryotic cells – in *E. faecalis* biofilm formation.

## Conclusion

The present study showed that a large portion of *E. faecalis* isolates from clinical samples form biofilms readily. The MLST of *E. faecalis* in this study demonstrated substantial ST diversity, with the main STs being ST16 and ST179. In general, biofilm formation was more strongly associated with ST16 than ST179. Our findings suggest a positive association between linezolid resistance in *E. faecalis* and robust biofilm formation. Interestingly, we found that the *cylA* gene was associated with weak biofilm formation of *E. faecalis* isolates, while the *esp* gene was only associated with strong or medium biofilm formation of *E. faecalis* isolates.

## Author Contributions

J-XZ participated in the design of the study, carried out the biofilm assay, analyzed and interpreted the data, and drafted the manuscript. YW participated in the analysis of the data and provided a critical revision of the manuscript. Z-WL conducted the RNA extraction, quantitative real-time PCR and the data analysis. Z-YP performed antibiotic-susceptibility testing, detected virulence genes by PCR, and participated in the data analysis. W-MY, ZC, and D-YL participated in the acquisition of the samples, isolated DNA, conducted MLST, and participated in the data analysis. Q-WD, DQ, and Z-JY designed the study, participated in the data analysis, and provided critical revisions of the manuscript for important intellectual content.

## Conflict of Interest Statement

The authors declare that the research was conducted in the absence of any commercial or financial relationships that could be construed as a potential conflict of interest.

## Footnotes

1 http://efaecalis.mlst.net/

## Abbreviations

Agg: aggregation substance
Asa1: surface aggregating protein
CI: confidence intervals
CLSI: Clinical and Laboratory Standards Institute
CylA: cytolysin A
eDNA: extracellular DNA
Esp: enterococcal surface protein
GelE: gelatinase
MIC: minimal inhibitory concentration
MLST: multilocus sequence typing
OR: Odds ratio
qRT-PCR: quantitative real-time polymerase chain reaction
ST: sequence type
VRE: vancomycin-resistant enterococci

## References

[B1] Anderson A. C. Jonas D. Huber I. Karygianni L. Wölber J. Hellwig E. (2016). *Enterococcus faecalis* from food, clinical specimens, and oral sites: prevalence of virulence factors in association with biofilm formation. *Front. Microbiol.* 6:1534 10.3389/fmicb.2015.01534 PMC4707231 26793174

[B2] Bonten M. J. Willems R. J. (2012). Vancomycin-resistant *Enterococcus*-chronicle of a foretold problem. *Ned. Tijdschr. Geneeskund.* 156 A5233. 22992249

[B3] Chuang-Smith O. N. Wells C. L. Henry-Stanley M. J. Dunny G. M. (2010). Acceleration of *Enterococcus faecalis* biofilm formation by aggregation substance expression in an ex vivo model of cardiac valve colonization. *PLOS ONE* 5:e15798. 10.1371/journal.pone.0015798 21209892 PMC3012704

[B4] Damborg P. Top J. Hendrickx A. P. Dawson S. Willems R. J. Guardabassi L. (2009). Dogs are a reservoir of ampicillin-resistant *Enterococcus faecium* lineages associated with human infections. *Appl. Environ. Microbiol.* 75 2360–2365. 10.1128/AEM.02035-08 19233953 PMC2675212

[B5] Di Rosa R. Creti R. Venditti M. D’Amelio R. Arciola C. R. Montanaro L. (2006). Relationship between biofilm formation, the enterococcal surface protein (Esp) and gelatinase in clinical isolates of *Enterococcus faecalis* and *Enterococcus faecium*. *FEMS Microbiol. Lett.* 256 145–150. 10.1111/j.1574-6968.2006.00112.x 16487332

[B6] Dupre I. Zanetti S. Schito A. M. Fadda G. Sechi L. A. (2003). Incidence of virulence determinants in clinical *Enterococcus faecium* and *Enterococcus faecalis* isolates collected in Sardinia (Italy). *J. Med. Microbiol.* 52 491–498. 10.1099/jmm.0.05038-0 12748268

[B7] Elhadidy M. Elsayyad A. (2013). Uncommitted role of enterococcal surface protein, Esp, and origin of isolates on biofilm production by *Enterococcus faecalis* isolated from bovine mastitis. *J. Microbiol. Immunol. Infect.* 46 80–84. 10.1016/j.jmii.2012.02.002 22520271

[B8] Fisher K. Phillips C. (2009). The ecology, epidemiology and virulence of *Enterococcus*. *Microbiology* 155 1749–1757. 10.1099/mic.0.026385-0 19383684

[B9] Flokas M. E. Karageorgos S. A. Detsis M. Alevizakos M. Mylonakis E. (2017). Vancomycin-resistant enterococci colonisation, risk factors and risk for infection among hospitalised paediatric patients: a systematic review and meta-analysis. *Int. J. Antimicrob. Agents* 49 565–572. 10.1016/j.ijantimicag.2017.01.008 28336313

[B10] Gawryszewska I. Żabicka D. Hryniewicz W. Sadowy E. (2017). Linezolid-resistant enterococci in Polish hospitals: species, clonality and determinants of linezolid resistance. *Eur. J. Clin. Microbiol. Infect. Dis.* 36 1279–1286. 10.1007/s10096-017-2934-7 28197728 PMC5495842

[B11] Kainer M. A. Devasia R. A. Jones T. F. Simmons B. P. Melton K. Chow S. (2007). Response to emerging infection leading to outbreak of linezolid-resistant enterococci. *Emerg. Infect. Dis.* 13 1024–1030. 10.3201/eid1307.070019 18214174 PMC2878236

[B12] Kayaoglu G. Ørstavik D. (2004). Virulence factors of *Enterococcus faecalis*: relationship to endodontic disease. *Crit. Rev. Oral Biol. Med.* 15 308–320. 10.1177/154411130401500506 15470268

[B13] Kozitskaya S. Olson M. E. Fey P. D. Witte W. Ohlse K. Ziebuhr W. (2005). Clonal analysis of *Staphylococcus epidermidis* isolates carrying or lacking biofilm-mediating genes by multilocus sequence typing. *J. Clin. Microbiol.* 43 4751–4757. 10.1128/JCM.43.9.4751-4757.2005 16145137 PMC1234069

[B14] Kristich C. J. Li Y. H. Cvitkovitch D. G. Dunny G. M. (2004). Esp independent biofilm formation by *Enterococcus faecalis*. *J. Bacteriol.* 186 154–163. 10.1128/JB.186.1.154-163.2004 14679235 PMC365672

[B15] Lebeaux D. Ghigo J. M. Beloina C. (2014). Biofilm-related infections: bridging the gap between clinical management and fundamental aspects of recalcitrance toward antibiotics. *Microbiol. Mol. Biol. Rev.* 78 510–543. 10.1128/MMBR.00013-14 25184564 PMC4187679

[B16] Manning S. D. Springman A. C. Lehotzky E. Lewis M. A. Whittam T. S. Davies H. D. (2009). Multilocus sequence types associated with neonatal group B streptococcal sepsis and meningitis in Canada. *J. Clin. Microbiol.* 47 1143–1148. 10.1128/JCM.01424-08 19158264 PMC2668308

[B17] Mohamed J. A. Huang W. Nallapareddy S. R. Teng F. Murray B. E. (2004). Influence of origin of isolates, especially endocarditis isolates, and various genes on biofilm formation by *Enterococcus faecalis*. *Infect. Immun.* 72 3658–3663. 10.1128/IAI.72.6.3658-3663.2004 15155680 PMC415661

[B18] Mohamed J. A. Murray B. E. (2005). Lack of correlation of gelatinase production and biofilm formation in a large collection of *Enterococcus faecalis* isolates. *J. Clin. Microbiol.* 43 5405–5407. 10.1128/JCM.43.10.5405-5407.2005 16208033 PMC1248489

[B19] Montealegre M. C. La Rosa S. L. Roh J. H. Harvey B. R. Murray B. E. (2015). The *Enterococcus faecalis* EbpA Pilus protein: attenuation of expression, biofilm formation, and adherence to fibrinogen start with the rare initiation codon ATT. *mBio.* 6:e00467-15. 10.1128/mBio.00467-15 26015496 PMC4447247

[B20] O’Driscoll T. Crank C. W. (2015). Vancomycin-resistant enterococcal infections: epidemiology, clinical manifestations, and optimal management. *Infect. Drug Resist.* 8 217–230. 10.2147/IDR.S54125 26244026 PMC4521680

[B21] Paganelli F. L. Willems R. J. Leavis H. L. (2012). Optimizing future treatment of enterococcal infections: attacking the biofilm? *Trends Microbiol.* 20 40–49. 10.1016/j.tim.2011.11.001 22169461

[B22] Park S. Y. Kim K. M. Lee J. H. Seo S. J. Lee I. H. (2007). Extracellular gelatinase of *Enterococcus faecalis* destroys a defense system in insect hemolymph and human serum. *Infect. Immun.* 75 1861–1869. 10.1128/IAI.01473-06 17261598 PMC1865674

[B23] Parker R. E. Laut C. Gaddy J. A. Zadoks R. Davies H. D. Manning S. (2016). Association between genotypic diversity and biofilm production in group B *Streptococcus*. *BMC Microbiol.* 16:86. 10.1186/s12866-016-0704-9 27206613 PMC4875601

[B24] Ruiz-Cruz S. Espinosa M. Goldmann O. Bravo A. (2015). Global regulation of gene expression by the MafR protein of *Enterococcus faecalis*. *Front. Microbiol.* 6:1521. 10.3389/fmicb.2015.01521 26793169 PMC4707282

[B25] Ruiz-Garbajosa P. Bonten M. Robinson D. Robinson A. Top J. Nallapareddy S. (2006). Multilocus sequence typing scheme for *Enterococcus faecalis* reveals hospital-adapted genetic complexes in a background of high rates of recombination. *J. Clin. Microbiol.* 44 2220–2228. 10.1128/JCM.02596-05 16757624 PMC1489431

[B26] Sandoe J. A. Witherden I. R. Cove J. H. Heritage J. Wilcox M. H. (2003). Correlation between enterococcal biofilm formation *in vitro* and medical-device-related infection potential *in vivo*. *J. Med. Microbiol.* 52 547–550. 10.1099/jmm.0.05201-0 12808074

[B27] Seno Y. Kariyama R. Mitsuhata R. Monden K. Kumon H. (2005). Clinical implications of biofilm formation by *Enterococcus faecalis* in the urinary tract. *Acta Med. Okayama* 59 79–87. 16049560 10.18926/AMO/31979

[B28] Sghir A. Gramet G. Suau A. Rochet V. Pochart P. Dore J. (2000). Quantification of bacterial groups within human fecal flora by oligonucleotide probe hybridization. *Appl. Environ. Microbiol.* 66 2263–2266. 10.1128/AEM.66.5.2263-2266.2000 10788414 PMC101487

[B29] Süßmuth S. D. Muscholl-Silberhorn A. Wirth R. Susa M. Marre R. Rozdzinski E. (2000). Aggregation substance promotes adherence, phagocytosis, and intracellular survival of *Enterococcus faecalis* within human macrophages and suppresses respiratory burst. *Infect. Immunity* 68 4900–4906. 10.1128/iai.68.9.4900-4906.2000 10948103 PMC101694

[B30] Tendolkar P. M. Baghdayan A. S. Gilmore M. S. Shankar N. (2004). Enterococcal surface protein, Esp, enhances biofilm formation by *Enterococcus faecalis*. *Infect. Immun.* 72 6032–6039. 10.1128/IAI.72.10.6032-6039.2004 15385507 PMC517584

[B31] Thomas V. C. Thurlow L. R. Boyle D. Hancock L. E. (2008). Regulation of autolysis-dependent extracellular DNA release by *Enterococcus faecalis* extracellular proteases influences biofilm development. *J. Bacteriol.* 190 5690–5698. 10.1128/JB.00314-08 18556793 PMC2519388

[B32] Toledo-Arana A. Valle J. Solano C. Arrizubieta M. Cucarella C. Lamata M. (2001). The enterococcal surface protein, Esp, is involved in *Enterococcus faecalis* biofilm formation. *Appl. Environ. Microbiol.* 67 4538–4545. 10.1128/AEM.67.10.4538-4545.2001 11571153 PMC93200

[B33] Treitman A. N. Yarnold P. R. Warren J. Noskin G. A. (2005). Emerging incidence of *Enterococcus faecium* among hospital isolates (1993 to 2002). *J. Clin. Microbiol.* 43 462–463. 10.1128/JCM.43.1.462-463.2005 15635016 PMC540171

[B34] Urwin R. Maiden M. C. (2003). Multi-locus sequence typing: a tool for global epidemiology. *Trends Microbiol.* 11 479–487. 10.1016/j.tim.2003.08.006 14557031

[B35] Van Harten R. M. Willems R. J. L. Martin N. I. Hendrickx A. P. A. (2017). Multidrug-resistant enterococcal infections: new compounds, novel antimicrobial therapies? *Trends Microbiol.* 25 467–479. 10.1016/j.tim.2017.01.004 28209400

[B36] Van Tyne D. Martin M. J. Gilmore M. S. (2013). Structure, function, and biology of the *Enterococcus faecalis* cytolysin. *Toxins* 5 895–911. 10.3390/toxins5050895 23628786 PMC3709268

[B37] Vankerckhoven V. Autgaerden T. Vael C. Lammens C. Chapelle S. Rossi R. (2004). Development of a multiplex PCR for the detection of asa1, gelE, cylA, esp, and hyl genes in enterococci and survey for virulence determinants among European hospital isolates of *Enterococcus faecium*. *J. Clin. Microbiol.* 42 4473–4479. 10.1128/JCM.42.10.4473-4479.2004 15472296 PMC522368

[B38] Weng P. L. Ramli R. Shamsudin M. N. Cheah Y. K. Hamat R. A. (2013). High genetic diversity of *Enterococcus faecium* and *Enterococcus faecalis* clinical isolates by pulsed-field gel electrophoresis and multilocus sequence typing from a hospital in Malaysia. *Biomed Res. Int.* 2013:938937. 10.1155/2013/938937 23819125 PMC3681219

[B39] Xu T. Wu Y. Lin Z. W. Bertram R. Götz F. Zhang Y. (2017). Identification of genes controlled by the essential YycFG two-component system reveals a role for biofilm modulation in *Staphylococcus epidermidis*. *Front. Microbiol.* 8:724. 10.3389/fmicb.2017.00724 28491057 PMC5405149

