# Supplementary material for: Characteristics of and Virulence Factors Associated with Biofilm Formation in Clinical Enterococcus faecalis Isolates in China
Source: Front Microbiol. 2017 Nov 24;8:2338. doi: 10.3389/fmicb.2017.02338 (PMC5705541; doi:10.3389/fmicb.2017.02338)
Supplement: Supplementary file 1 [file Table_1.DOC]

**Table S1. Biofilm formation according to ST of 224 *E. faecalis* isolates.**

| **STs (n)** | **No. (%) of isolates with biofilm phenotype** | | | | |
| --- | --- | --- | --- | --- | --- |
|  | **Strong** | **Medium** | ***Strong or medium*** | **Weak** | **Positive for biofilm formation** |
| ST4 (3) | 0 (0.0 ) | 1 (33.3 ) | *1 (33.3 )* | 2 (66.7 ) | 3 (100.0 ) |
| ST6 (2) | 0 (0.0 ) | 0 (0.0 ) | *0 (0.0 )* | 1 (50.0 ) | 1 (50.0 ) |
| ST11 (4) | 0 (0.0 ) | 0 (0.0 ) | *0 (0.0 )* | 2 (50.0 ) | 2 (50.0 ) |
| ST16 (79) | 19 (24.1 ) | 9 (11.4 ) | *28 (35.4 )* | 20 (25.3 ) | 48 (60.8.8 ) |
| ST19 (1) | 0 (0.0 ) | 0 (0.0 ) | *0 (0.0 )* | 0 (0.0 ) | 0 (0.0 ) |
| ST21 (2) | 0 (0.0 ) | 0 (0.0 ) | *0 (0.0 )* | 0 (0.0 ) | 0 (0.0 ) |
| ST22 (1) | 0 (0.0 ) | 0 (0.0 ) | *0 (0.0 )* | 0 (0.0 ) | 0 (0.0 ) |
| ST28 (1) | 0 (0.0 ) | 0 (0.0 ) | *0 (0.0 )* | 1 (100.0 ) | 1 (100.0 ) |
| ST30 (9) | 0 (0.0 ) | 0 (0.0 ) | *0 (0.0 )* | 0 (0.0 ) | 0 (0.0 ) |
| ST34 (1) | 0 (0.0 ) | 0 (0.0 ) | *0 (0.0 )* | 0 (0.0 ) | 0 (0.0 ) |
| ST40 (4) | 3 (75.0 ) | 0 (0.0 ) | *3 (75.0 )* | 0 (0.0 ) | 3 (75.0 ) |
| ST41 (2) | 0 (0.0 ) | 0 (0.0 ) | *0 (0.0 )* | 0 (0.0 ) | 0 (0.0 ) |
| ST44 (2) | 0 (0.0 ) | 0 (0.0 ) | *0 (0.0 )* | 2 (100.0 ) | 2 (100.0 ) |
| ST47 (2) | 1 (50.0 ) | 1 (50.0 ) | *2 (100.0 )* | 0 (0.0 ) | 2 (100.0 ) |
| ST63 (1) | 0 (0.0 ) | 0 (0.0 ) | *0 (0.0 )* | 1 (100.0 ) | 1 (100.0 ) |
| ST64 (1) | 0 (0.0 ) | 0 (0.0 ) | *0 (0.0 )* | 0 (0.0 ) | 0 (0.0 ) |
| ST67 (1) | 0 (0.0 ) | 0 (0.0 ) | *0 (0.0 )* | 0 (0.0 ) | 0 (0.0 ) |
| ST69 (1) | 1 (100.0 ) | 0 (0.0 ) | *1 (100.0 )* | 0 (0.0 ) | 1 (100.0 ) |
| ST79 (2) | 0 (0.0 ) | 0 (0.0 ) | *0 (0.0 )* | 1 (50.0 ) | 1 (50.0 ) |
| ST100 (1) | 0 (0.0 ) | 1 (100.0 ) | *1 (100.0 )* | 0 (0.0 ) | 1 (100.0 ) |
| ST126 (1) | 0 (0.0 ) | 0 (0.0 ) | *0 (0.0 )* | 0 (0.0 ) | 0 (0.0 ) |
| ST139 (1) | 0 (0.0 ) | 1 (100.0 ) | *1 (100.0 )* | 0 (0.0 ) | 1 (100.0 ) |
| ST143 (1) | 0 (0.0 ) | 0 (0.0 ) | *0 (0.0 )* | 0 (0.0 ) | 0 (0.0 ) |
| ST179 (68) | 1 (1.5 ) | 3 (4.4 ) | *4 (5.9 )* | 24 (35.3 ) | 28 (41.2 ) |
| ST191 (2) | 2 (100.0 ) | 0 (0.0 ) | *2 (100.0 )* | 0 (0.0 ) | 2 (100.0 ) |
| ST202 (1) | 0 (0.0 ) | 0 (0.0 ) | *0 (0.0 )* | 0 (0.0 ) | 0 (0.0 ) |
| ST207 (1) | 0 (0.0 ) | 0 (0.0 ) | *0 (0.0 )* | 1 (100.0 ) | 1 (100.0 ) |
| ST300 (1) | 0 (0.0 ) | 0 (0.0 ) | *0 (0.0 )* | 0 (0.0 ) | 0 (0.0 ) |
| ST314 (2) | 0 (0.0 ) | 1 (50.0 ) | *1 (50.0 )* | 0 (0.0 ) | 1 (50.0 ) |
| ST387 (2) | 0 (0.0 ) | 0 (0.0 ) | *0 (0.0 )* | 1 (50.0 ) | 1 (50.0 ) |
| ST403 (3) | 0 (0.0 ) | 0 (0.0 ) | *0 (0.0 )* | 0 (0.0 ) | 0 (0.0 ) |
| ST409 (4) | 0 (0.0 ) | 1 (25.0 ) | *1 (25.0 )* | 0 (0.0 ) | 1 (25.0 ) |
| ST410 (1) | 1 (100.0 ) | 0 (0.0 ) | *1 (100.0 )* | 0 (0.0 ) | 1 (100.0 ) |
| ST414 (1) | 0 (0.0 ) | 0 (0.0 ) | *0 (0.0 )* | 0 (0.0 ) | 0 (0.0 ) |
| ST436 (1) | 0 (0.0 ) | 0 (0.0 ) | *0 (0.0 )* | 1 (100.0 ) | 1 (100.0 ) |
| ST474 (1) | 0 (0.0 ) | 0 (0.0 ) | *0 (0.0 )* | 0 (0.0 ) | 0 (0.0 ) |
| ST476 (1) | 0 (0.0 ) | 0 (0.0 ) | *0 (0.0 )* | 0 (0.0 ) | 0 (0.0 ) |
| ST480 (4) | 3 (75.0 ) | 1 (25.0 ) | *4 (100.0 )* | 0 (0.0 ) | 4 (100.0 ) |
| ST506 (1) | 0 (0.0 ) | 0 (0.0 ) | *0 (0.0 )* | 0 (0.0 ) | 0 (0.0 ) |
| ST541 (3) | 0 (0.0 ) | 1 (33.3 ) | *1 (33.3 )* | 2 (66.7 ) | 3 (100.0 ) |
| ST581 (1) | 0 (0.0 ) | 0 (0.0 ) | *0 (0.0 )* | 0 (0.0 ) | 0 (0.0 ) |
| ST585 (1) | 1 (100.0 ) | 0 (0.0 ) | *1 (100.0 )* | 0 (0.0 ) | 1 (100.0 ) |
| ST619 (1) | 0 (0.0 ) | 0 (0.0 ) | *0 (0.0 )* | 0 (0.0 ) | 0 (0.0 ) |
| ST7480 (1) | 1 (100.0 ) | 0 (0.0 ) | *1 (100.0 )* | 0 (0.0 ) | 1 (100.0 ) |
| Total (224) | 33 (14.7 ) | 20 (8.9 ) | *53 (23.6 )* | 59 (26.3 ) | 112 (50.0 ) |
