# Supplementary material for: Characteristics of and Virulence Factors Associated with Biofilm Formation in Clinical Enterococcus faecalis Isolates in China
Source: Front Microbiol. 2017 Nov 24;8:2338. doi: 10.3389/fmicb.2017.02338 (PMC5705541; doi:10.3389/fmicb.2017.02338)
Supplement: Supplementary file 2 [file Table_2.DOC]

**Table S2.** **Prevalence of ST16 and ST179 within clinical *E. faecalis* isolates by source.**

| **Clinical source (n)** | **N (%)** | |
| --- | --- | --- |
|  | **ST16** | **ST179** |
| Blood (21) | 10 (47.6) | 7 (33.3) |
| Urine (98) | 34 (34.7) | 32 (32.7) |
| Pus or Secretions (48) | 15 (31.3) | 18 (37.5) |
| Bile (12) | 4 (33.3) | 1 (8.3) |
| Other (45) | 16 (35.6) | 10 (22.2) |
| Total (224) | 79 (35.3) | 68 (30.4) |
