# Supplementary material for: Characteristics of and Virulence Factors Associated with Biofilm Formation in Clinical Enterococcus faecalis Isolates in China
Source: Front Microbiol. 2017 Nov 24;8:2338. doi: 10.3389/fmicb.2017.02338 (PMC5705541; doi:10.3389/fmicb.2017.02338)
Supplement: Supplementary file 3 [file Table_3.DOC]

**Table S3.** **Prevalence of ST16 and ST179 within linezolid-sensitivity groups of *E. faecalis* isolates.**

| **ST** | **N (%) in each level of linezolid MIC in µg/mL** | | |
| --- | --- | --- | --- |
|  | **MIC ≤ 2**  **N = 180** | **2 < MIC < 8**  **N = 36** | **8 ≤ MIC**  **N = 8** |
| ST16 | 61 (33.9) | 16 (44.4) | 2 (25.0) |
| ST179 | 58 (32.2) | 10 (27.8) | 0 (0.0) |
